# Endocrine and exocrine pancreatic insufficiency after acute pancreatitis: long-term follow-up study

**DOI:** 10.1186/s12876-017-0663-0

**Published:** 2017-10-27

**Authors:** Jianfeng Tu, Jingzhu Zhang, Lu Ke, Yue Yang, Qi Yang, Guotao Lu, Baiqiang Li, Zhihui Tong, Weiqin Li, Jieshou Li

**Affiliations:** 1 Research Institute of General Surgery, Jinling Hospital, Medical School of Nanjing University, 305 East Zhongshan Road, Nanjing, 210002 China; 2 Zhejiang Provincial People’s Hospital, People’s Hospital of Hangzhou Medical College, Shangtang road 158#, Hangzhou, 310014 China; 3 Hangzhou Medical College, Binwen road 481#, Hangzhou, 310053 China

**Keywords:** Endocrine pancreatic insufficiency, Exocrine pancreatic insufficiency, Acute pancreatitis, Follow-up study, Insulin resistance, Pancreatic necrosis

## Abstract

**Background:**

Patients could develop endocrine and exocrine pancreatic insufficiency after acute pancreatitis (AP), but the morbidity, risk factors and outcome remain unclear. The aim of the present study was to evaluate the incidence of endocrine and exocrine pancreatic insufficiency after AP and the risk factors of endocrine pancreatic insufficiency through a long-term follow-up investigation.

**Methods:**

Follow-up assessment of the endocrine and exocrine function was conducted for the discharged patients with AP episodes. Oral Glucose Tolerance Test (OGTT) and faecal elastase-1(FE-1) test were used as primary parameters. Fasting blood-glucose (FBG), fasting insulin (FINS), glycosylated hemoglobin HBA1c, 2-h postprandial blood glucose (2hPG), Homa beta cell function index (HOMA-β), homeostasis model assessment of insulin resistance (HOMA-IR) and FE-1 were collected. Abdominal contrast-enhanced computed tomography (CECT) was performed to investigate the pancreatic morphology and the other related data during hospitalization was also collected.

**Results:**

One hundred thirteen patients were included in this study and 34 of whom (30.1%) developed diabetes mellitus (DM), 33 (29.2%) suffered impaired glucose tolerance (IGT). Moreover, 33 patients (29.2%) developed mild to moderate exocrine pancreatic insufficiency with 100μg/g<FE-1<200μg/g and 7 patients (6.2%) were diagnosed with severe exocrine pancreatic insufficiency with FE-1<100μg/g. The morbidity of DM and IGT in patients with pancreatic necrosis was significant higher than that in the non-pancreatic necrosis group (*X* ^*2*^ = 13.442,*P* = 0.001). The multiple logistic regression analysis showed that extent of pancreatic necrosis<30% (*P* = 0.012, OR = 0.061) were the protective factors of endocrine pancreatic insufficiency. HOMA-IR (*P* = 0.002, OR = 6.626), Wall-off necrosis (WON) (*P* = 0.013, OR = 184.772) were the risk factors.

**Conclusion:**

The integrated morbidity of DM and IGT after AP was 59.25%, which was higher than exocrine pancreatic insufficiency. 6.2% and 29.2% of patients developed severe and mild to moderate exocrine pancreatic insufficiency, respectively. The extent of pancreatic necrosis>50%, WON and insulin resistance were the independent risk factors of new onset diabetes after AP.

**Electronic supplementary material:**

The online version of this article (10.1186/s12876-017-0663-0) contains supplementary material, which is available to authorized users.

## Background

Patients could develop endocrine and exocrine pancreatic insufficiency after AP, but the morbidity, risk factors, treatment and outcome remain unclear. The most controversial part is about the risk factors of endocrine pancreatic insufficiency. Das et al. [1] reported that prediabetes and diabetes were common after AP with about 40% prevalence. Reccurent attacks, hyperglycaemia, obesity, age above 45 years, family history of DM were the risk factors,but severity of AP showed minimal effect on it. Hsiu-Nien Shen et al. found that the overall risk of DM increased by two-fold after the first-attack of AP and the risk of diabetes for mild AP patients were similar to those for all AP [2]. However,other studies suggested that the severity of AP was a risk factor of the DM after AP [3, 4]. But it was the insufficient of these studies with small size and short follow-up time. In the present study,we conduct a long-term follow-up investigation to assess the incidence of endocrine and exocrine pancreatic insufficiency after AP attacks and the risk factors of endocrine pancreatic insufficiency.

## Methods

### Patients

From January to April 2016, this study was undertaken in the sever acute pancreatitis(SAP) care center of Nanjing University, which is one of the largest SAP centers in China. One hundred twenty four discharged patients in our outpatients database were randomly invited to the hospital to participate in the follow-up study by phone or mail. The written informed consent was obtained from each subject. The study was approved by the ethics committee of the Jinling Hospital, Medical School of Nanjing University.

The exclusion criteria were as follows: I. Patients who suffered recurrent AP; II. Patients with chronic pancreatitis; III. Patients with diagnosed DM before AP episodes; IV. Patients suffered from chronic diarrhea before AP; V. Patients with intestinal tuberculosis or Crohn’s disease; VI. Patients with family history of DM; VII. Patients with incomplete medical record. VIII. Patients who died during hospitalization or after discharge from hospital.

### Assessment methods and data collection:

Simplified OGTT [5] and FE-1 test were applied to assess the endocrine and exocrine pancreatic function. The value of FBG, FINS, HBA1C, 2hPG, HOMA-β, HOMA-IR and FE-1 from the two tests were collected as evaluation indexes. Abdominal CECT was performed for pancreatic morphology. The stool samples were collected and stored in −20 °C for FE-1 test. The symptoms such as abdomen pain, diarrhea, diet, exercise, medication were inquired and recorded. The other information of each patient during their hospitalization such as onset time, admission time, discharge time, diagnosis time for DM or IGT, family history of DM, smoking and alcoholism history, Etiology, the classification of AP, APACHE II score [6], Balthazar score [7], systemic complications such as Acute Kidney Injury (AKI), Acute Respiratory Distress Syndrome (ARDS), etc., local complications (pancreatic infection, pancreatic necrosis, etc.); location and extent of pancreatic necrosis from CT scan image, treatment such as percutaneous catheter drainage (PCD), Operative Necrosectomy, etc. were also collected.

### Evaluation index

Endocrine pancreatic function index included DM symptoms (polydipsia, polyphagia, urorrhagia, loss of weight, etc.), FBG, FINS, Fasting c-peptide, HBA1C, 2hPG. The HOMA-β which represents the function of β-cell and HOMA-IR which represents the condition of insulin resistance were respectively calculated by the formula of [HOMA-β = 20 × FINS/(FPG-3.5)] [8] and [HOMA-IR = FPG × FINS/22.5] [9]. Exocrine pancreatic function index included the symptoms of exocrine pancreatic insufficiency (abdominal pain, abdominal distension, diarrhea, fat diarrhea, etc.), value of FE-1 and blood albumin.

### Definition

#### Diabetes

Diabetes was defined using the 1999 World Health Organization criteria. It was diagnosed by Typical diabetes symptoms with any of the following items:

A. FPG ≥ 7.0 mmol/L.
B. Random blood glucose ≥ 11.1 mmol/L.
C. FPG<7.0 mmol/L and 2hPG>11.1 mmol/L after a 75-g OGTT.

Diabetes was also diagnosed by any of the following items if without classical diabetes symptom:

A. FPG>7.0 mmol/L for 2 times.
B. 2hPG ≥ 11.1 mmol/L for 2 times.

#### Igt

IGT was diagnosed by FPG<7.0 mmol/L and 7.8 mmol/L<2hPG<11.1 mmol/L after a 75-g OGTT.

#### Exocrine pancreatic insufficiency

FE-1 test (BIOSERV Diagnostics GmbH, Rostock, Germany) was used to assess the exocrine pancreatic function. Reference concentration for FE-1 in stool was as follows:

- Normal exocrine pancreatic function: above 200μg/g stool,
- mild to moderate exocrine pancreatic function: 100 to 200μg/g stool,
- severe exocrine pancreatic function: less than 100μg/g stool [10, 11].

### Statistical analysis:

Statistical analysis was performed using SPSS 22.0 for Windows (SPSS Inc., Chicago, Ill). Non-parametric tests were used to analyze the data. When comparing more than 3 groups, the Kruskal-Wallis test was used. Comparison between 2 groups was made with Mann-Whitney U test. The *X* ^*2*^ test was used to compare categorical variables. Fisher test was used when expected frequencies were less than 5. Multiple logistic regression analysis was used to analysis the risk factors of endocrine pancreatic insufficiency. Odds ratios (ORs) are expressed with 95% confidence intervals (CIs).A *P* value of<0.05 was considered significant.

## Results

### General information

Finally, 113 patients were included and 11 patients were excluded due to meeting the exclusion criteria, change of address or declining to participating in the study. Among the 11 cases, 7 patients (5.6% in all patients) died during hospitalization or after discharge from hospital due to different reasons(4 for septic shock, 2 for major bleeding and 1 died out of hospital for unknown reason). Of the 113 eligible patients, there were 75 male and 38 female with a mean age of 47.2 ± 1.3 years (median, 46 years). The shortest interval from the AP onset to follow-up assessment was 1 month and the longest was 260 months with a mean value of 42.93 ± 4.03 months (median, 30 months). 83.2% patients were first episode. For the severity, 10 patients (8.8%) were classified as Mild AP (MAP), 12 patients (10.6%) as Moderate Severe AP (MSAP) and the remaining 91 patients (80.6%) were all diagnosed as Severe AP(SAP). The detail data was listed in the Tables 1 and 2.

**Table 1:** General characteristics of the patients with AP (1)

Variable	$\overline{X}$	S.E.	Median	Minimum	Maximum	Percentile25	Percentile75
Age(year)	47.2	1.3	46.0	13.0	80.0	38.5	54.0
Time Interval(month)	42.93	4.03	30	1.0	260.0	10.0	66.0
APACHE II	9.24	0.64	7.0	0	32	4.0	13.0
Balthazar Score	6.83	0.25	8.0	1.0	10.0	5.0	9.5
Recurrence Rate	1.51	0.19	1.0	1.0	20.0	1.0	1.0

Time Interval, the time from AP onset to follow-up visit; APACHE II, Acute Physiology and Chronic Health Evaluation II

**Table 2:** General characteristics of the patients with AP (2)

Variable	N	%
Sex		
Male	75	66.4
Female	38	33.6
Classification		
MAP	10	8.8
MSAP	12	10.6
SAP	91	80.6
Etiology		
Biliary	65	57.5
HTG	39	34.5
Alcoholic	3	2.7
Others	6	5.3
ARDS		
Mild	23	20.4
Moderate	20	17.7
Severe	15	13.3
No	55	48.7
AKI		
AKI-I	13	11.5
AKI-II	12	10.6
AKI-III	23	20.4
No	65	57.5
Pancreatic Necrosis		
Yes	89	78.8
No	24	21.2
WON		
Yes	7	6.2
No	106	93.8
Pancreatic Infection		
Yes	73	64.6
No	40	35.4
Part of Pancreatic Necrosis		
Head of pancreas	11	12.36
Body of pancreas	12	13.48
Tail of pancreas	51	57.3
Whole pancreas	15	16.85
Area of Pancreatic Necrosis		
<1/3	31	34.83
1/3–50%	26	35.96
>50%	89	29.21
PCD		
Yes	81	71.7
No	32	28.3
ON		
Yes	32	28.3
No	81	71.7
Morphology of Pancreas		
Absence or atrophy of the Head of Pancreas	17	15.0
Absence or atrophy of the Body and/or tail of Pancreas	40	35.4
Absence or atrophy of the whole pancreas	11	9.7
Normal area of pancreas	45	39.8

*HTG* hypertriglyceridemia, *WON* wall-off necrosis, *PCD* percutaneous catheter drainage, *ON* operative necrosectomy; Morphology of Pancreas, outline of pancreas by CT scan at follow-up time

### Morbidity of endocrine and exocrine pancreatic insufficiency

**Fig. 1:**
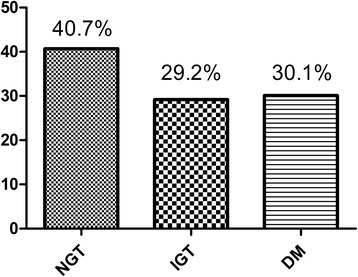
Morbidity of endocrine pancreatic insufficiency

**Fig. 2:**
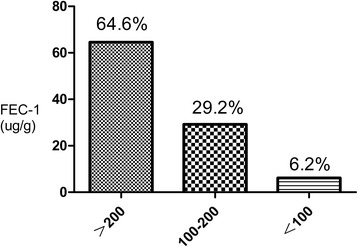
Morbidity of exocrine pancreatic insufficiency

Thirty four of 113 patients (30.1%) was diagnosed with DM, 33 patients (29.2%) with IGT and 46 patients (40.7%) with normal endocrine function as shown in Fig. 1. The incidence of abdominal pain, abdominal distension and diarrhea (including fat diarrhea) was respectively 5.3%, 10.6% and 15.04%. Body Mass Index (BMI) of 4.4% study subjects was lower than 18. Seventy three patients (64.6%), 33 patients (29.2%) and 7 patients (6.2%) were defined as normal, mild to moderate and severe exocrine pancreatic function, respectively as shown in Fig. 2.

### Comparison of endocrine and exocrine pancreatic function between the patients with different follow-up time interval

According to the time interval from the AP onset to follow-up assessment, the patients were divided into 3 groups, respectively as “group<3 months”, “group 3 months-5 years” and “group>5 years”. The morbidity of endocrine pancreatic insufficiency and the value of FE-1 among the 3 groups showed no significant difference (*X* ^2^ = 4.751,*P* = 0.235 and *X* ^2^ = 3.262, *P* = 0.515, respectively). The difference regarding the value of HBA1C among the 3 groups was also no significant (*X* ^2^ = 0.731, *P* = 0.484). The detail data was listed in the Table 3.

**Table 3:** Comparison of endocrine and exocrine pancreatic function between the different time interval groups

	<3 m (*N* = 9, 7.9%)	3 m-5y (*N* = 75, 66.4%)	>5y (*N* = 29,25.7%)	X^2^/F Value	*P* Value
Endocrine function				4.751	0.235*
DM	22.2%	25.3%	44.8%		
IGT	44.4%	29.3%	24.1%		
NGT	33.3%	45.3%	31.1%		
HOMA-β(%)(X ± S.E.)	78.81 ± 15.23	80.31 ± 6.13	66.82 ± 8.92	0.731	0.484
FE-1				3.262	0.515*
>200	66.7%	66.7%	58.6%		
100–200	33.3%	25.3%	37.9%		
<100	0	8%	3.4%		

*IGT* impaired glucose tolerance, *NGT* normal glucose tolerance, *FE-1* faecal elastase-1; * Fish Exact Test

### Endocrine and exocrine pancreatic function of patients with different location and extent of pancreatic necrosis

According to the ECET images, the patients were divided into group pancreatic necrosis and group non-pancreatic necrosis. The morbidity of DM and IGT in patients with pancreatic necrosis was significant higher than group non-pancreatic necrosis (*X* ^2^ = 13.442,*P* = 0.001). The value of FE-1 between the 2 groups showed no significant difference (*X* ^2^ = 0.242,*P* = 0.886)as listed in Table 4. The cases were also divided into group necrosis area<30%,group 50%>necrosis area>30% and group necrosis area>50% on the basis of different extent of pancreatic necrosis. The morbidity of DM and IGT and the value of FE-1 between the 3 groups showed no significant difference. But the value of HBA1C (*X* ^*2*^ = 7.525, *P* = 0.001) and HOMA-β (*X* ^*2*^ = 13.088, *P* = 0.000) among the 3 groups were significantly different as shown in Table 5. According to the CECT images, group pancreatic necrosis was divided into 4 sub-groups again, such as group head of pancreas, group body of pancreas, group tail of pancreas and group whole pancreas. The value of HOMA-β (*X* ^2^ = 5.173, *P* = 0.002) and the morbidity of DM and IGT (*X* ^2^ = 12.79, *P* = 0.046) in group tail of pancreas and group whole pancreas was significant different with the other 2 groups. But it showed no significant difference in the value of FE-1 between 4 sub-groups (*X* ^2^ = 3.267, *P* = 0.775) as listed in Table 6.

**Table 4:** Comparison of endocrine and exocrine pancreatic function between group pancreatic necrosis and group non-pancreatic necrosis

	Pancreatic Necrosis (*n* = 89,78.8%)	Non- Pancreatic Necrosis (*n* = 24, 21.2%)	F/X^2^ Value	*P* Value
Endocrine function			13.442	0.001
NGT	34.8%	62.5%		
IGT	27%	37.5%		
DM	38.2%	0		
FE-1			0.242	0.886
>200	64.1%	66.6%		
100–200	29.2%	29.2%		
<100	6.7%	4.2%

**Table 5:** Comparison of endocrine and exocrine pancreatic function between the different area of pancreatic necrosis groups

	<30%	30%–50%	>50%	X^2^/F Value	*P* Value
Endocrine function				8.957	0.062
NGT	45.2%	34.4%	23.1%		
IGT	35.5%	25.0%	19.2%		
DM	19.4%	40.6%	57.7%		
HBA1C%(HPLC) (X ± S.E.)	5.54 ± 0.32	5.69 ± 0.11	6.57 ± 0.27	7.525	0.001
HOMA-β(%) (X ± S.E.)	101.65 ± 10.12	60.65 ± 6.91	43.54 ± 6.60	13.088	0.000
FE-1				4.435	0.35
>200	67.7%	71.9%	50.0%		
100–200	22.6%	25.0%	42.3%		
<100	9.7%	3.1%	7.7%

**Table 6:** Comparison of endocrine and exocrine pancreatic function between the different part of pancreatic necrosis groups

	Head of Pancreas	Body of Pancreas	Tail of Pancreas	Whole Pancreas	F/X^2^ Value	*P* Value
Endocrine function					12.79	0.046
NGT	63.6%	50.0%	29.4%	20.0%		
IGT	18.2%	41.7%	23.5%	33.3%		
DM	18.2%	8.3%	47.1%	46.7%		
HOMA-β(%)(X ± S.E.)	100.16 ± 15.42	104.44 ± 19.42	61.34 ± 6.11	49.39 ± 9.11	5.173	0.002
FE-1					3.267	0.775
>200	54.5%	75.0%	60.8%	73.3%		
100–200	36.4%	25.0%	31.4%	20.0%		
<100	9.1%	0	7.8%	6.7%		

### Endocrine and exocrine pancreatic function of patients with pancreatic infection and different AP classification.

The morbidity of DM and IGT in patients with pancreatic infection was significant higher than those without (*X* ^2^ = 9.139,*P* = 0.01). But the difference of the value of FE-1 between the 2 groups was not significant (*X* ^2^ = 0.29, *P* = 0.865) as shown in Table 7. According to the Atlanta criteria, 113 patients were divided into group MAP (*n* = 10, 8.9%), group MSAP (*n* = 12, 10.6%) and group SAP (*n* = 91, 80.5%). Both the morbidity of DM and IGT (*X* ^2^ = 8.439, *P* = 0.069) and the value of FE-1 (*X* ^2^ = 1.272, *P* = 0.906) between 3 groups was no significant difference as listed in Table 8.

**Table 7:** Comparison of endocrine and exocrine pancreatic function between the group pancreatic infection and group non-pancreatic infection

	Pancreatic Infection (*n* = 73, 64.6%)	Non-Pancreatic Infection(*n* = 40,35.4%)	F/X^2^ Value	*P* Value
Endocrine function			9.139	0.01
NGT	35.6%	50%		
IGT	24.7%	37.5%		
DM	39.7%	12.5%		
FE-1			0.29	0.865
>200	63.0%	67.5%		
100–200	30.1%	27.5%		
<100	6.8%	5.0%

**Table 8:** Comparison of endocrine and exocrine pancreatic function between the different AP classification

	(N = 10, 8.9%)	MSAP (*N* = 12, 10.6%)	SAP (*N* = 91, 80.5%)	X^2^/F Value	*P* Value
DM Morbidity				8.439	0.069
NGT	70%	58.33%	35.16%		
IGT	30%	25%	29.67%		
DM	0	16.67%	35.16%		
FEC-1				1.272	0.906
>200	80%	66.67%	62.64%		
100–200	20%	33.33%	29.67%		
<100	0	0	7.69%		

### Risk factors analyzed by multiple logistic regression analysis

These factors such as sex, age, part and area of pancreatic necrosis, pancreatic infection et al. were included into the logistic regression analysis according the above mentioned results and clinical characteristics. The results showed that male (*P* = 0.01, OR = 0.083), 18–44 years age (*P* = 0.018, OR = 0.018), PCD (*P* = 0.001,OR = 0.006), necrosis of the head of the pancreas (*P* = 0.007, OR = 0.009), extent of pancreatic necrosis<30% (*P* = 0.012, OR = 0.061) was the protective factors of endocrine pancreatic insufficiency. HOMA-IR (*P* = 0.002, OR = 6.626) and WON (*P* = 0.013, OR = 184.772) were the risk factors as shown in detail in Table 9.

**Table 9:** Risk factors of endocrine pancreatic insufficiency by multiple logistic regression analysis

	Wald	P	Exp(B)	95% C.I. lower	95% C.I. upper
Sex(male)	6.616	0.01	0.083	0.012	0.553
Age	13.532	0.001			
age(18-44y)	5.583	0.018	0.018	0.001	0.506
age(45-64y)	0.012	0.913	1.153	0.091	14.646
HOMA-IR	9.666	0.002	6.626	2.011	21.825
PCD(yes)	10.636	0.001	0.006	0.000	0.134
WON(yes)	6.195	0.013	184.772	3.032	11,258.328
Part of pancreatic necrosis	11.779	0.008			
Head of pancreas	7.290	0.007	0.009	0.000	0.27
Body of pancreas	3.698	0.054	0.045	0.002	1.061
Tail of pancreas	0.066	0.798	0.746	0.080	6.994
Pancreatic infection(yes)	2.843	0.328	1.237	0.067	11.215
Area of pancreatic necrosis	7.154	0.028			
<30%	6.276	0.012	0.024	0.001	0.446
30%–50%	5.819	0.016	0.061	0.006	0.592
AKI(No)	3.741	0.291			
AKI-1	0.038	0.845	0.428	0.037	4.889
AKI-2	0.066	0.797	6.887	1.206	3.331
AKI-3	3.419	0.064	2.851	0.028	1.359

## Discussion

A few patients will develop endocrine and exocrine pancreatic insufficiency after recovering from AP episodes, which catch more and more attention than before in recent years as more patients survive from severe AP. In traditional opinion, disturbance of carbohydrate metabolism should resulted from acute stress, pancreatic microcirculation disorder and excessive secretion of catecholamine after AP, which leading to transient rising in blood glucose. After the improvement of disease, the blood glucose will return to normal soon [12–14]. But part of the patients could not fully recover from the hyperglycemia in the end and some patients’ blood glucose could rise again after a short time recovery. Some patients even develop DM and need treatment with antidiabetic or insulin in their rest of lives [15, 16]. In our study, DM and IGT occurred in 34 and 33 of the study patients respectively. Symersky assessed the endocrine pancreatic function of patients who recovered from AP and found out 32% MAP patients and 42% SAP patients still suffered from disturbance of carbohydrate metabolism. He also suggested that patients who received pancreas surgery had higher risk of glucose metabolism disorder [13]. However, the risk factors of endocrine pancreatic insufficiency were controversial and need further verification.

The diagnosis of the new-onset diabetes after AP was not unified and usually confused by type2 diabetes mellitus. But the World Health Organization and American Diabetes Association has defined it as “pancreatogenic diabetes” and classified it as a form type 3c diabetes mellitus (T3c DM) with a prevalence of 5–10% among all diabetic subjects in western population [17–20]. About 80% of T3cDM patients were diagnosed as a complication of chronic pancreatitis. Acute pancreatitis, pancreatic cancer, pancreatectomy et al. are the other common causes of T3cDM [21, 22]. Thus studies about pathomechanism of T3cDM mostly focused on chronic pancreatitis. Persistent chronic inflammation of the pancreatic tissue in patients with chronic pancreatitis could lead to pancreatic fibrosis and islet damage both of which result in islet β-cell insufficiency, hepatic insulin resistance and finally occurrence of DM [23, 24].

Compare to endocrine pancreatic insufficiency, exocrine pancreatic insufficiency is more difficult to diagnose. Usually the symptoms such as abdominal pain, abdominal extension and fat diarrhea combined with radiological examination and stool test are used for precise diagnosis [25, 26]. In our study, 5.3%, 10.6% and 15.04% of patients respectively suffered from abdominal pain, abdominal extension and diarrhea (including fat diarrhea) after discharge. The BMI of 4.4% of patients was lower than 18. Thus it can be seen that the symptom of exocrine pancreatic insufficiency is neither usual nor specific which is of less value for diagnosis. In contrast, FE-1 was much better with relatively high stability and specificity and was verified to be a good indirect index of exocrine pancreatic insufficiency by a few studies [27, 28]. We found 6.2% of patients could be diagnosed with severe exocrine pancreatic insufficiency (<100μg/g)and 29.2% of patients only showed mild to moderate (100-200μg/g) insufficiency. There are some scholars doubt it’s specificity and sensitivity. Leeds found that FE-1<100μg/g was highly specific for exocrine pancreatic insufficiency, however 100-200μg/g could only offer limited specificity and sensitivity [29]. On the other hand, we couldn’t know the patients’ baseline value of FE-1 before AP and the stool sample preparation is complicated. So the diagnosis of exocrine pancreatic insufficiency by FE-1 should be strengthened by other diagnostic tools such as MRI of pancreatic duct [30].

In our study, the morbidity of DM and IGT showed no significant difference between the different time interval groups. But we also found that as time goes on, the value of HBA_1_C gradually increased in the study patients. This phenomenon suggests that endocrine pancreatic function could weaken over time. But we could not confirm if it resulted from the disease or the natural course. Therefore, more long-term studies with larger sample size is needed to verify the role of time interval from onset of AP to follow-up time.

It is reported in previous studies that the disease severity of AP had no relationship with new-onset diabetes [1, 2]. We also found that the morbidity of endocrine and exocrine pancreatic insufficiency among group MAP, group MSAP and group SAP was not significantly different. But pancreatic necrosis, which is an important marker for disease severity, was found as an independent risk factor in multiple logistic regression analysis. We also found the difference of the disease severity indexes and complications between group NGT, group IGT and group DM as detailed in Additional file 1: Tables S1-S2 of the additional file. Compare to the DM after pancreatectomy, large scale pancreatic necrosis may has similar pathogenesis to secondary diabetes which could also lead to great decline in the number of β-cell and insulin secretion [31–33]. Garip reported that the patients with SAP, pancreatectomy and pancreatic necrosis especially those with large extent of necrosis had higher risk of endocrine pancreatic insufficiency than patients with MAP [14]. The significant difference of pancreatic necrosis between the three groups was revealed as listed in Table A3 of the additional file. Above all, we may could not simply deny the effect of disease severity on the endocrine pancreatic insufficiency. Pancreatic necrosis may play an important role in the new-onset diabetes.

We also observe that female, age>45 years, pancreatic necrosis, extent of pancreatic necrosis>50%, WON, insulin resistance are the independent risk factors of endocrine pancreatic insufficiency, while PCD is the protective factor. For the age, it is recognized that prevalence of DM increase exponentially after 45 years of age [33, 34]. But Hsiu-Nien Shen et al. found that the highest age-specific HR of DM was observed in men aged<45 years (HR = 7.46) [2]. So according to the current research outcome, we couldn’t affirm the effect of gender and age and it needs more studies to verify.

## Conclusions

The integrated morbidity of DM and IGT after AP was 59.25%, which was much higher than that of exocrine pancreatic insufficiency. Only 6.2% and 29.2% of patients respectively developed severe exocrine pancreatic insufficiency and mild to moderate exocrine pancreatic insufficiency in the present study. Pancreatic necrosis, extent of pancreatic necrosis>50%, WON and insulin resistance were the independent risk factors of new onset diabetes after AP. For the diagnosis of exocrine pancreatic insufficiency, the FE-1 test is easy, but still not an ideal evaluation index for exocrine pancreatic function.

## Additional file

Additional file 1: Tables S1-S3. Comparision on the disease severity, complication and pancreatic necrosis between NGT, IGT and DM groups. The APACHE II score and Balthazar score in group DM was significant higher than that in group IGT and group NGT (X^2^ = 5.257, *P* = 0.007; X^2^ = 13.03, *P* = 0.000). The value of the HOMA-IR in group DM and group IGT was significant higher than group NGT (X^2^ = 4.025, *P* = 0.021). Morbidity of AKI in group DM was higher than in group IGT and group NGT (F = 20.885, *P* = 0.001), but the complication of ARDS in 3 groups showed no significant difference(X^2^ = 4.453, *P* = 0.627). Compare to group IGT and group NGT, the morbidity of pancreatic necrosis in group DM was significant higher and 100% patients in group DM got pancreatic necrosis (X^2^ = 13.442, *P* = 0.001). For pancreatic necrosis, the proportion of the tail of pancreas and whole pancreas during hospitalization in group DM was higher than other two groups (X^2^ = 11.788, *P* = 0.063, likely attributed to type II error). The area of pancreatic necrosis>50% and the area<1/3 in group DM was higher and lower than in group IGT and group NGT respectively (X^2^ = 8.957, *P* = 0.062, likely attributed to type II error). The atrophy or absence of the body and tail of pancreas in group DM at follow-up time was significant more than the other two groups (X^2^ = 43.92, *P* = 0.000). The morbidity of pancreatic infection in group DM was also showed much higher than group IGT and group NGT (X^2^ = 9.139, *P* = 0.01). (DOCX 31 kb)

## Abbreviations

2hPG: 2-h postprandial blood glucose
AKI: Acute kidney injury
AP: Acute pancreatitis
ARDS: Acute respiratory distress syndrome
BMI: Body mass index
CECT: Contrast-enhanced computed tomography
DM: Diabetes mellitus
FBG: Fasting blood-glucose
FE-1: Faecal elastase-1
FINS: Fasting insulin
HOMA-IR: Homeostasis model assessment of insulin resistance
HOMA-β: Homa beta cell function index
IGT: Impaired glucose tolerance
MAP: Mild AP
MSAP: Moderate Severe AP
OGTT: Oral Glucose Tolerance Test
PCD: Percutaneous catheter drainage
SAP: Sever acute pancreatitis
WON: Wall-off necrosis
