# Supplementary material for: Endocrine and exocrine pancreatic insufficiency after acute pancreatitis: long-term follow-up study
Source: BMC Gastroenterol. 2017 Oct 27;17:114. doi: 10.1186/s12876-017-0663-0 (PMC5658961; doi:10.1186/s12876-017-0663-0)
Supplement: Additional file 1: Tables S1-S3. — Comparision on the disease severity, complication and pancreatic necrosis between NGT, IGT and DM groups. The APACHE II score and Balthazar score in group DM was significant higher than that in group IGT and group NGT (X2 = 5.257, P = 0.007; X2 = 13.03, P = 0.000). The value of the HOMA-IR in group DM and group IGT was significant higher than group NGT (X2 = 4.025, P = 0.021). Morbidity of AKI in group DM was higher than in group IGT and group NGT (F = 20.885, P = 0.001), but the complication of ARDS in 3 groups showed no significant difference(X2 = 4.453, P = 0.627). Compare to group IGT and group NGT, the morbidity of pancreatic necrosis in group DM was significant higher and 100% patients in group DM got pancreatic necrosis (X2 = 13.442, P = 0.001). For pancreatic necrosis, the proportion of the tail of pancreas and whole pancreas during hospitalization in group DM was higher than other two groups (X2 = 11.788, P = 0.063, likely attributed to type II error). The area of pancreatic necrosis>50% and the area<1/3 in group DM was higher and lower than in group IGT and group NGT respectively (X2 = 8.957, P = 0.062, likely attributed to type II error). The atrophy or absence of the body and tail of pancreas in group DM at follow-up time was significant more than the other two groups (X2 = 43.92, P = 0.000). The morbidity of pancreatic infection in group DM was also showed much higher than group IGT and group NGT (X2 = 9.139, P = 0.01). (DOCX 31 kb) [file 12876_2017_663_MOESM1_ESM.docx]

**Table**

**Table 1 General characteristics of the patients with AP (1)**

| **Variable** | $\bar{\boldsymbol{X}\bar{\boldsymbol{X}}}$ | **S.E.** | **Median** | **Minimum** | **Maximum** | **Percentile25** | **Percentile75** |
| --- | --- | --- | --- | --- | --- | --- | --- |
| Age(year)  Time Interval(month)  APACHE II  Balthazar Score  Recurrence Rate | 47.2  42.93  9.24  6.83  1.51 | 1.3  4.03  0.64  0.25  0.19 | 46.0  30  7.0  8.0  1.0 | 13.0  1.0  0  1.0  1.0 | 80.0  260.0  32  10.0  20.0 | 38.5  10.0  4.0  5.0  1.0 | 54.0  66.0  13.0  9.5  1.0 |
| Time Interval, the time from AP onset to follow-up visit; APACHE II, Acute Physiology and Chronic Health Evaluation II. | | | | | | | |

**Table 2 General characteristics of the patients with AP (2)**

| **Variable** | **N** | **%** |
| --- | --- | --- |
| Sex  Male  Female  Classification  MAP  MSAP  SAP  Etiology  Biliary  HTG  Alcoholic  Others  ARDS  Mild  Moderate  Severe  No  AKI  AKI-I  AKI-II  AKI-III  No  Pancreatic Necrosis  Yes  No  WON  Yes  No  Pancreatic Infection  Yes  No  Part of Pancreatic Necrosis  Head of pancreas  Body of pancreas  Tail of pancreas  Whole pancreas  Area of Pancreatic Necrosis  ＜1/3  1/3-50%  ＞50%  PCD  Yes  No  ON  Yes  No  Morphology of Pancreas  Absence or atrophy of the Head of Pancreas  Absence or atrophy of the Body and/or tail of Pancreas  Absence or atrophy of the whole pancreas  Normal area of pancreas | 75  38  10  12  91  65  39  3  6  23  20  15  55  13  12  23  65  89  24  7  106  73  40  11  12  51  15  31  26  89    81  32  32  81  17  40  11  45 | 66.4  33.6  8.8  10.6  80.6  57.5  34.5  2.7  5.3  20.4  17.7  13.3  48.7  11.5  10.6  20.4  57.5  78.8  21.2  6.2  93.8  64.6  35.4  12.36  13.48  57.3  16.85  34.83  35.96  29.21  71.7  28.3  28.3  71.7  15.0  35.4  9.7  39.8 |

HTG, hypertriglyceridemia; WON, wall-off necrosis; PCD, percutaneous catheter drainage; ON, operative necrosectomy; Morphology of Pancreas, outline of pancreas by CT scan at follow-up time.

|  |
| --- |

**Table 3 Comparison of endocrine and exocrine pancreatic function between the different time interval groups**

|  | **＜3m**  **(N=9, 7.9%)** | **3m-5y**  **(N=75, 66.4%)** | **＞5y**  **(N=29,25.7%)** | **X^2^/F Value** | **P Value** |
| --- | --- | --- | --- | --- | --- |
| Endocrine function  DM  IGT  NGT | 22.2%  44.4%  33.3% | 25.3%  29.3%  45.3% | 44.8%  24.1%  31.1% | 4.751 | 0.235* |
| HOMA-β(%)(X±S.E.) | 78.81±15.23 | 80.31±6.13 | 66.82±8.92 | 0.731 | 0.484 |
| FE-1  ＞200  100-200  ＜100 | 66.7%  33.3%  0 | 66.7%  25.3%  8% | 58.6%  37.9%  3.4% | 3.262 | 0.515* |
| IGT, impaired glucose tolerance; NGT, normal glucose tolerance; FE-1, faecal elastase-1; * Fish Exact Test | | | | | |

**Table 4 Comparison of endocrine and exocrine pancreatic function between group pancreatic necrosis and group non-pancreatic necrosis**

|  | **Pancreatic**  **Necrosis (n=89，78.8%)** | **Non- Pancreatic**  **Necrosis (n=24, 21.2%)** | **F/X^2^ Value** | **P Value** |
| --- | --- | --- | --- | --- |
| Endocrine function  NGT  IGT  DM  FE-1  ＞200  100-200  ＜100 | 34.8%  27%  38.2%  64.1%  29.2%  6.7% | 62.5%  37.5%  0  66.6%  29.2%  4.2% | 13.442  0.242 | 0.001  0.886 |

**Table 5 Comparison of endocrine and exocrine pancreatic function between the different area of pancreatic necrosis groups**

|  | **＜30%** | **30%-50%** | **＞50%** | | **X^2^/F Value** | **P Value** |
| --- | --- | --- | --- | --- | --- | --- |
| Endocrine function  NGT  IGT  DM  HBA1C%(HPLC)  (X±S.E.)  HOMA-β(%)(X±S.E.)  FE-1  ＞200  100-200  ＜100 | 45.2%  35.5%  19.4%  5.54±0.32  101.65±10.12  67.7%  22.6%  9.7% | 34.4%  25.0%  40.6%  5.69±0.11  60.65±6.91  71.9%  25.0%  3.1% | | 23.1%  19.2%  57.7%  6.57±0.27  43.54±6.60  50.0%  42.3%  7.7% | 8.957  7.525  13.088  4.435 | 0.062  0.001  0.000  0.35 |

**Table 6 Comparison of endocrine and exocrine pancreatic function between the different part of pancreatic necrosis groups**

|  | **Head of Pancreas** | **Body of Pancreas** | **Tail of Pancreas** | **Whole Pancreas** | **F/X^2^ Value** | **P Value** |
| --- | --- | --- | --- | --- | --- | --- |
| Endocrine function  NGT  IGT  DM  HOMA-β(%)(X±S.E.)  FE-1  ＞200  100-200  ＜100 | 63.6%  18.2%  18.2%  100.16±15.42  54.5%  36.4%  9.1% | 50.0%  41.7%  8.3%  104.44±19.42  75.0%  25.0%  0 | 29.4%  23.5%  47.1%  61.34±6.11  60.8%  31.4%  7.8% | 20.0%  33.3%  46.7%  49.39±9.11  73.3%  20.0%  6.7% | 12.79  5.173  3.267 | 0.046  0.002  0.775 |

**Table 7 Comparison of endocrine and exocrine pancreatic function between the group pancreatic infection and group non-pancreatic infection**

|  | **Pancreatic Infection**  **(n=73, 64.6%)** | **Non-Pancreatic Infection(n=40,35.4%)** | **F/X^2^ Value** | **P Value** |
| --- | --- | --- | --- | --- |
| Endocrine function  NGT  IGT  DM  FE-1  ＞200  100-200  ＜100 | 35.6%  24.7%  39.7%  63.0%  30.1%  6.8% | 50%  37.5%  12.5%  67.5%  27.5%  5.0% | 9.139  0.29 | 0.01  0.865 |

**Table 8 Comparison of endocrine and exocrine pancreatic function between the different AP classification**

|  | **MAP**  **(N=10, 8.9%)** | **MSAP**  **(N=12, 10.6%)** | **SAP**  **(N=91, 80.5%)** | **X^2^/F Value** | **P Value** |
| --- | --- | --- | --- | --- | --- |
| DM Morbidity  NGT  IGT  DM  FEC-1  ＞200  100-200  ＜100 | 70%  30%  0  80%  20%  0 | 58.33%  25%  16.67%  66.67%  33.33%  0 | 35.16%  29.67%  35.16%  62.64%  29.67%  7.69% | 8.439  1.272 | 0.069  0.906 |

**Table 9 Risk factors of endocrine pancreatic insufficiency by** **multiple logistic regression analysis**

|  | **Wald** | **P** | **Exp(B)** | **95% C.I. lower** | **95% C.I.**  **upper** |
| --- | --- | --- | --- | --- | --- |
| Sex（male）  Age  age（18-44y）  age（45-64y）  HOMA-IR  PCD（yes）  WON（yes）  Part of pancreatic necrosis  Head of pancreas  Body of pancreas  Tail of pancreas  Pancreatic infection（yes）  Area of pancreatic necrosis  ＜30%  30%-50%  AKI（No）  AKI-1  AKI-2  AKI-3 | 6.616  13.532  5.583  0.012  9.666  10.636  6.195  11.779  7.290  3.698  0.066  2.843  7.154  6.276  5.819  3.741  0.038  0.066  3.419 | 0.01  0.001  0.018  0.913  0.002  0.001  0.013  0.008  0.007  0.054  0.798  0.328  0.028  0.012  0.016  0.291  0.845  0.797  0.064 | 0.083  0.018  1.153  6.626  0.006  184.772  0.009  0.045  0.746  1.237  0.024  0.061  0.428  6.887  2.851 | 0.012  0.001  0.091  2.011  0.000  3.032  0.000  0.002  0.080  0.067  0.001  0.006  0.037  1.206  0.028 | 0.553  0.506  14.646  21.825  0.134  11258.328  0.27  1.061  6.994  11.215  0.446  0.592  4.889  3.331  1.359 |
